# The genetic basis of isolated mitochondrial complex II deficiency

**DOI:** 10.1016/j.ymgme.2020.09.009

**Published:** 2020

**Authors:** Millie Fullerton, Robert McFarland, Robert W. Taylor, Charlotte L. Alston

**Affiliations:** a Wellcome Centre for Mitochondrial Research, Newcastle University, Framlington Place, Newcastle upon Tyne, NE2 4HH, UK; b Translational and Clinical Research Institute, Faculty of Medical Sciences, Newcastle University, Newcastle upon Tyne, NE2 4HH, UK; c NHS Highly Specialised Services for Rare Mitochondrial Disorders, Royal Victoria Infirmary, Newcastle upon Tyne Hospitals NHS Foundation Trust, Queen Victoria Road, Newcastle upon Tyne, NE1 4LP, UK

**Keywords:** Mitochondrial disease, Complex II, Succinate dehydrogenase, Pathogenic variants

## Abstract

Mitochondrial complex II (succinate:ubiquinone oxidoreductase) is the smallest complex of the oxidative phosphorylation system, a tetramer of just 140 kDa. Despite its diminutive size, it is a key complex in two coupled metabolic pathways - it oxidises succinate to fumarate in the tricarboxylic acid cycle and the electrons are used to reduce FAD to FADH_2_, ultimately reducing ubiquinone to ubiquinol in the respiratory chain. The biogenesis and assembly of complex II is facilitated by four ancillary proteins, all of which are autosomally-encoded. Numerous pathogenic defects have been reported which describe two broad clinical manifestations, either susceptibility to cancer in the case of single, heterozygous germline variants, or a mitochondrial disease presentation, almost exclusively due to bi-allelic recessive variants and associated with an isolated complex II deficiency. Here we present a compendium of pathogenic gene variants that have been documented in the literature in patients with an isolated mitochondrial complex II deficiency. To date, 61 patients are described, harbouring 32 different pathogenic variants in four distinct complex II genes: three structural subunit genes (*SDHA, SDHB* and *SDHD*) and one assembly factor gene (*SDHAF1*). Many pathogenic variants result in a null allele due to nonsense, frameshift or splicing defects however, the missense variants that do occur tend to induce substitutions at highly conserved residues in regions of the proteins that are critical for binding to other subunits or substrates. There is phenotypic heterogeneity associated with defects in each complex II gene, similar to other mitochondrial diseases.

## 1 Introduction

Mitochondria are subcellular organelles that are present in all nucleated cells, participating in many cellular pathways including calcium signalling and apoptosis. However, it is their role in the production of cellular energy, in the form of adenosine triphosphate (ATP), that dominates their function, earning them the moniker the ‘powerhouse of the cell’ [1]. Over 90% of all adenosine triphosphate is generated by the mitochondrial oxidative phosphorylation (OXPHOS) system located within the inner mitochondrial membrane. In healthy individuals, this aerobic respiration provides the cell with sufficient energy to undertake its metabolic activities. Under normal conditions, electrons are transferred via a series of redox reactions across four multimeric respiratory chain complexes situated within the inner mitochondrial membrane - NADH:ubiquinone oxidoreductase (complex I), succinate:ubiquinone oxidoreductase (complex II), ubiquinol:cytochrome *c* oxidoreductase (complex III) and cytochrome *c* oxidase (complex IV) - and protons are concomitantly pumped from the matrix into the intermembrane space to create an electrochemical gradient [2]. The mitochondrial ATP synthase (Complex V) utilises this electrochemical gradient to drive the phosphorylation of ADP to generate ATP [2]. This review focuses on mammalian complex II and serves to summarise current knowledge pertaining to the structure and function of the complex, together with a comprehensive review of the pathogenic genetic variants identified in patients with primary mitochondrial disease presentations, briefly touching on the role of defective complex II in tumorigenesis.

## 2 The structure and assembly of succinate dehydrogenase

Unlike other mitochondrial respiratory chain complexes, complex II is encoded entirely by autosomal genes; complexes I, III, IV and V all include structural subunits encoded by the mitochondria's own genetic material – mtDNA [3]. Complex II is the smallest complex in the OXPHOS system, being composed of just four structural subunits encoded by genes sharing the same name: *SDHA* (5p15.33; OMIM *600857,Genbank: NM_004168.4), *SDHB* (1p36.13, OMIM *185470, Genbank: NM_003000.3), *SDHC* (1q23.3, OMIM *602413, Genbank NM_003001.3) and *SDHD* (11q23.1, OMIM *602690, Genbank NM_003002.4) [4,5]. In order to achieve the specific quaternary structure of complex II, four assembly factors (SDHAF1-4) are required to coordinate its biogenesis; these are encoded by the *SDHAF1* (19q13.12, OMIM *612848, Genbank: NM_001042631.2), *SDHAF2* (11q12.2, OMIM *613019, Genbank: NM_017841.2), *SDHAF3* (7q21.3, OMIM *615773, Genbank: NM_020186.2) and *SDHAF4* (6q13, Genbank: NM_145267.3) genes. Collectively, the genes encoding the structural subunits and assembly factors are referred to as “*SDHx*” genes.

**Fig. 1:**
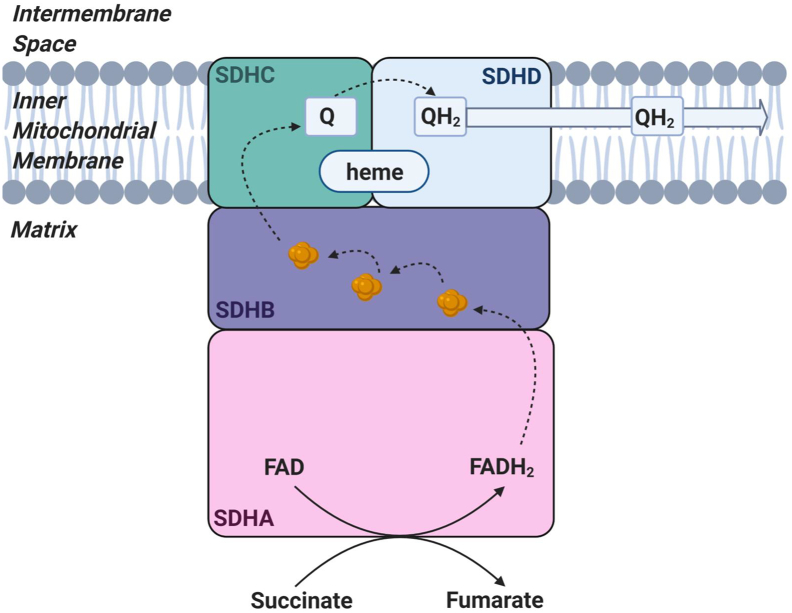
Schematic illustrating the structure and function of complex II. The catalytic flavoprotein subunit (SDHA) protrudes into the matrix, where it functions to oxidise succinate to fumarate and reduces FAD to FADH_2_. The electrons released from this reaction are shuttled through the Fe-S clusters (shown in orange) through SDHB to reduce the electron carrier ubiquinone (Q) to produce ubiquinol (QH_2_) which passes the electrons from complex II to complex III. Electron transfer is indicated by arrows.

Despite its diminutive size, the complex is crucial for two metabolic pathways. The catalytic core, formed by the flavoprotein subunit SDHA and the iron‑sulphur cluster protein SDHB, protrudes into the matrix where it oxidises succinate to fumarate within the tricarboxylic acid (TCA) cycle (Fig. 1). The structure also comprises complex II of the respiratory chain where the SDHA-SDHB heterodimer conjoins with the integral membrane proteins SDHC and SDHD that are embedded within the inner mitochondrial membrane. The largest subunit, SDHA, has a molecular weight of 70 kDa and contains a covalently-attached flavin adenine dinucleotide (FAD) cofactor. It also harbours a succinate binding site, where succinate is oxidised to fumarate within the tricarboxylic acid cycle [4]. SDHB, a 27 kDa iron-sulphur protein, contains three iron-sulphur clusters that accept the two electrons generated from succinate oxidation and pass them to ubiquinone within the inner mitochondrial membrane for translocation down the OXPHOS system to complex III [4]. Unlike the other complexes of the mitochondrial respiratory chain, complex II does not contribute to the proton gradient that drives ADP phosphorylation by ATP synthase (complex V) [2]. In addition to the FAD cofactor, SDH also harbours a prosthetic heme *b* between the integral membrane-bound subunits SDHC and SDHD, and a ubiquinone binding site. The heme *b* prosthetic group is embedded between SDHC and SDHD through conserved histidine residues; its precise role is yet undetermined although it is not directly involved in electron transfer from FAD to ubiquinone, nor does it play a significant role in ROS suppression or production [6]. Instead, studies of the planarity of the porphyrin ring in *Escherichia coli* heme *b* has provided evidence of structural heterogeneity, with a change in state related to the redox state of ubiquinone *in vivo* thereby proposing a role for heme *b* in molecular signalling [7].

Following translation on cytosolic ribosomes, each nascent subunit of complex II is translocated to the mitochondrial matrix via the TIM/TOM import systems [8] before folding, cofactor incorporation and finally assembly into the SDH holoenzyme [9]. Extensive studies in *Saccharomyces cerevisiae* have revealed the precise stages of SDH assembly and these have been reviewed elsewhere [10]. The three iron-sulphur clusters of complex II: [2Fe-2S], [3Fe-4S] and [4Fe-4S] [11], are generated by mitochondrial machinery including Frataxin, NSD11, ISCU and NFS1 which are then transferred to the Fe-S scaffold protein complex comprised of HSC20, HSPA9 and ISCU; the Fe-S clusters are inserted into SDHB through the interaction with the assembly factor SDHAF1 (previously known as LYRM8) which recruits the HSC20-HSPA9-ISCU iron‑sulphur scaffold via its LYR motifs [10,12,13]. SDHAF2 has been shown to stimulate the covalent binding of the FAD cofactor within SDHA. SDHAF3 has been postulated to protect the exposed iron-sulphur clusters within SDHB from reactive oxygen species during the maturation process [14]. Finally, SDHAF4 serves as a chaperone to facilitate SDHA and SDHB heterodimer formation [10].

## 3 The involvement of complex II defects in human pathology

Pathogenic variants affecting complex II genes result in mitochondrial dysfunction. This can manifest either as a primary mitochondrial disease associated with recessively inherited germline defects, or as susceptibility to tumorigenesis in the case of somatic mutation. We will briefly summarise the role of *SDHx* gene defects in inherited cancer before focusing on the clinical manifestations associated with the inborn error of metabolism.

### 3.1 Pathogenic *SDHx* variants and cancer

Multiple pathomechanisms link *SDHx* variants and tumorigenesis, including increased ROS production, defective apoptosis and the abnormal stabilization of hypoxia-inducible factors (HIF), all of which fit with established models of tumorigenesis [15]. Pathogenic variants in *SDHB* and *SDHC* in particular, have been associated with the development of paragangliomas (PGLs) and pheochromocytomas (PHEOs); these are usually benign catecholamine-secreting tumours that are derived from the chromaffin cells of the adrenal medulla in the case of PGL, and the extra adrenal ganglia in the case of PHEOs [16]. Although approximately 75% of incidences of PGL/PCC remain localised and do not metastasize, they remain a cause of mortality and morbidity consequent to hypertension from uncontrolled catecholamine production and secretion. The association between *SDHx* defects and tumour development is not limited to PGL and PHEOs, but extends to other solid tissue tumours including gastrointestinal stromal tumours (GISTs), pituitary adenomas and renal cell carcinomas [15]. Where there is a hereditary component, patients are typically found to have inherited a single heterozygous pathogenic variant through the germline; in accordance with Knudson's theory, the second pathogenic ‘hit’ occurs as a random mutational event over the individual's lifetime and results in bi-allelic inactivation of SDH in that cell which evolves into a tumour through clonal expansion [17]. Although patients presenting with an isolated complex II deficiency harbour bi-allelic germline complex II gene defects, these are typically fatal during childhood, before the typical age of onset for these cancers. Although it would be expected that carrier parents of clinically-affected children must have a genetic predisposition for tumour manifestation given they are carriers of a pathogenic *SDHx* variant [18], there is no data to support this in the family histories studied here. Despite this, clinical guidelines recommend surveillance of these at risk individuals to facilitate early detection of any abnormalities [19]. The remaining part of this review will focus on primary mitochondrial disease presentations associated with complex II dysfunction; the current knowledge of the link between complex II defects and cancer has been comprehensively reviewed elsewhere [15].

### 3.2 *SDHx* defects and primary mitochondrial disease

Mitochondrial complex II deficiency is a rare inborn error of metabolism, accounting for approximately 2% of mitochondrial disease diagnoses [20]*.* In order to identify all genetic variants associated with complex II deficiency, and their associated clinical phenotypes, a comprehensive search of the available literature was performed culminating in a catalogue of all pathogenic variants identified in either a structural subunit or a known assembly factor of complex II. To ensure that all patients reported with confirmed mitochondrial complex II disease consequence to *SDHx* defects were collated, the following key terms were used to search on PubMed (https://www.ncbi.nlm.nih.gov/pubmed): “mitochondria”, “complex II deficiency”, “*SDHA*”, “*SDHB*”, “*SDHC*”, “*SDHD*”, “*SDHAF1*”, “*SDHAF2*”, “*SDHAF3*”, “*SDHAF4*”, “pathogenic variants”, “mutations”. Articles were restricted to those written in English; where manuscripts were identified in other languages, attempts were made to obtain appropriate clinical information from the authors to facilitate incorporation in the review. All mitochondrial complex II deficiency review articles were cross referenced to ensure that all cases have been captured. A total of 21 separate reports were identified, describing 61 clinically-affected patients and 32 different pathogenic *SDHx* variants; the genetic and phenotypic data from this comprehensive literature review are presented in Table 1.

**Table 1:** Clinical symptoms and molecular genetic diagnoses for all complex II deficient cases.

Case	Gene	cDNA	Predicted consequence	M/F	Consan-guinity	Ethnicity	Family history	Age of onset	Dev delay	Dev regression	Cardio-myopathy	Dys-tonia	Hypo-tonia	Ataxia	OA	Other	Patient ID	Refs
1	*SDHA*	c.1351C>T (het)	p.(Arg451Cys)	F	No	Caucasian	No	46 y	−	−	−	−	−	+	+	Double vision; limited ocular movements; visual loss; vertigo; headaches; aphonia; backache; proximal weakness	P1	[22]
2	*SDHA*	c.1351C>T (het)	p.(Arg451Cys)	F	No	Caucasian	No	mid-40s	−	−	−	−	−	+	+	Headaches; dizziness; blackouts; visual loss; dysesthesia; nystagmus	P2	[22]
3	*SDHA*	c.1351C>T (het)	p.(Arg451Cys)	M	No	Caucasian	No	15 y	+	+	+	−	−	+	+	Ocular paresis; cramps; pyramidal signs; cardiomegaly; nystagmus	P1	[23]
4	*SDHA*	c.1351C>T (het)	p.(Arg451Cys)	M	No	Caucasian	Yes	8 m	−	−	+	−	−	−	+	Gastroenteritis	P2	[23]
5	*SDHA*	c.1351C>T (het)	p.(Arg451Cys)	F	No	Caucasian	Yes			−	+	−	−	−		Died age 7 m	P3	[23]
6	*SDHA*	c.1A>C	Start loss	F	No		No	9 m	+	+	−			+		MRI: necrotic lesions in basal ganglia; Leigh syndrome	P1	[20]
		c.1571C>T	p.(Ala524Val)															
7	*SDHA*	c.248C>T	p.(Ala83Val) / p.(Ala83_Gln104del)	F	No	Caucasian	No	5 m	+	+	−	−	+	−		Hypsarrhythmia; tetraspasticity; visual loss; nystagmus; unable to speak; MRI: changes in caudate nucleus; globus pallidus; putamen	P1	[17], [24]
		c.356G>A	p.(Trp119*)															
8	*SDHA*	c.91C>T	p.(Arg31*)		No	Caucasian	No	birth	+	+	−	−		−		Seizures; apnoea	P2	[17]
		c.565T>G	p.(Cys189Gly)															
9	*SDHA*	c.117del	p.(Asn40Thrfs*18)	M	No	Chinese	No	4 y	+	+	−	−	−	−		Leigh-like syndrome; MRI: bilateral signal abnormalities in globus pallidus	P1	[25]
		c.221dup	p.(Leu74Phefs*9)															
10	*SDHA*	c.771_772del	p.(Gly258Leufs*62)		Yes			4 m		+		−	−			Microcephaly; spasticity; MRS: elevated succinate and lactate; MRI thalami and midbrain changes; died age 3y(respiratory failure)	P14	[26]
		c.1065_1260del	p.(Gly356Serfs*2)															
11	*SDHA*	c.1065-3C>A (hom)	splicing		Yes	Pakistani	No	4 w	+	−	−	−		−		Leukodystrophy; seizures; died age 2y 9 m	P3	[17]
12	*SDHA*	c.64-2A>G (hom)	splicing		No	Caucasian	No	16 m	+	+	+	−		+		Chorea; tremor	P4	[17]
13	*SDHA*	c.1664G>A (hom)	p.(Gly555Glu)	F	Yes	Bedouin		2 m			+					Respiratory distress	A6	[27]
14	*SDHA*	c.1664G>A (hom)	p.(Gly555Glu)	M	Yes	Bedouin		8 m	−		+						A7	[27]
15	*SDHA*	c.1664G>A (hom)	p.(Gly555Glu)	F	Yes	Bedouin		4 m	−		+					Respiratory distress	A8	[27]
16	*SDHA*	c.1664G>A (hom)	p.(Gly555Glu)	M	Yes	Bedouin		2 m			+					Respiratory distress	A9	[27]
17	*SDHA*	c.1664G>A (hom)	p.(Gly555Glu)	F	Yes	Bedouin		3 m			+					Respiratory distress	A10	[27]
18	*SDHA*	c.1664G>A (hom)	p.(Gly555Glu)	F	Yes	Bedouin		2 m			+					Respiratory distress	A11	[27]
19	*SDHA*	c.1664G>A (hom)	p.(Gly555Glu)	M	Yes	Bedouin		5 m	−							Mild respiratory distress	A12	[27]
20	*SDHA*	c.1664G>A (hom)	p.(Gly555Glu)	M	Yes	Bedouin		6 m			+					Respiratory distress	A13	[27]
21	*SDHA*	c.1664G>A (hom)	p.(Gly555Glu)	M	Yes	Bedouin		Prenatal			+					Respiratory distress	B1	[27]
22	*SDHA*	c.1664G>A (hom)	p.(Gly555Glu)	F	Yes	Bedouin		1 m	−							Respiratory distress	B2	[27]
23	*SDHA*	c.1664G>A (hom)	p.(Gly555Glu)	F	Yes	Bedouin		3 m			+					Respiratory distress	B3	[27]
24	*SDHA*	c.1664G>A (hom)	p.(Gly555Glu)	F	Yes	Bedouin		Prenatal			+					Respiratory distress	B4	[27]
25	*SDHA*	c.1664G>A (hom)	p.(Gly555Glu)	M	Yes	Bedouin		Prenatal	−		+					Respiratory distress	B5	[27]
26	*SDHA*	c.1664G>A (hom)	p.(Gly555Glu)	M	Yes	Bedouin		4 m			+					Respiratory distress	C1	[27]
27	*SDHA*	c.1664G>A (hom)	p.(Gly555Glu)	M	Yes	Bedouin		8 m	−							Respiratory distress	D1	[27]
28	*SDHA*	c.1664G>A (hom)	p.(Gly555Glu)	M	Yes	Palestinian	No	22 m	+	+	−	+	−			Spasticity; nystagmus; tetraparesis; MRI: bilateral signal abnormalities involving posterior and medial thalami, pons and medulla	P1	[28]
29	*SDHA*	c.1664G>A (hom)	p.(Gly555Glu)	F	Yes	Middle Eastern		5 m	−	−	+	−	+	−		Fever; rhinitis; wheezing; hepatosplenomegaly; cardiomegaly	P1	[29]
30	*SDHA*	c.1660C>T (hom)	p.(Arg554Trp)	F	Yes	Algerian	No	10 m		+	−						P1	[21]
31	*SDHA*	c.1660C>T (hom)	p.(Arg554Trp)	F	Yes	Algerian	Yes	10 m			−						P2	[21]
32	*SDHA*	c.1523C>T	p.(Thr508Ile)	M	No		No	3 m	+	−	+	−	+	−	−	Hypertonia; dyspnoea; feeding difficulties	P1	[31]
		c.1526C>T	p.(Ser509Leu)															
33	*SDHB*	c.143A>T (hom)	p.(Asp48Val)	F	Yes	Asian	No	12 m	+	+			+			Flexion contractures; MRI: dystrophic white matter	P2	[31]
34	*SDHB*	c.143A>T (hom)	p.(Asp48Val)	F	Yes	Pakistani	No	15 m	+				+			Central conduction abnormalities; hyperreflexia; frequent crying	P1	[34]
35	*SDHB*	c.143A>T (hom)	p.(Asp48Val)		Yes			6 m	+	+		−	+			Limb spasticity; severe motor difficulties; severe cognitive impairment	P16	[26]
36	*SDHB*	c.143A>T (hom)	p.(Asp48Val)		Yes			15 m		+		−	+	−		Hyperreflexia; marked irritability; moderate cognitive impairment	P19	[26]
37	*SDHB*	c.143A>T (hom)	p.(Asp48Val)	M	Yes	Turkish	No	Birth	+				+	+		Spasticity	LD756	[41]
38	*SDHB*	c.143A>T (hom)	p.(Asp48Val)		Yes			18 m		+		+		+		Spastic tetraparesis; normal cognition	P10	[26]
39	*SDHB*	c.143A>T (hom)	p.(Asp48Val)		No			12 m	+	+	+	+		+		Gross motor impairment with contractures; normal cognition	P11	[26]
40	*SDHB*	c.143A>T	p.(Asp48Val)	M	No	Turkish & Swedish	No	6 m	+	+	+	−	+			Strabismus; anisometropia; sudden relapse and died age 1y from multiorgan failure	P2	[35]
		c.689G>A	p.(Arg230His)															
41	*SDHB*	c.143A>T	p.(Asp48Val)		No			Birth		+		−				Died of respiratory failure age 1y	P15	[26]
		c.308 T>C	p.(Met103Thr)															
42	*SDHB*	c.304G>A (hom)	p.(Ala102Thr)	M	No	Indian	Yes	18 m	+	+					+	Seizures; increased tone in all four limbs; deep tendon reflexes	P1	[37]
43	*SDHB*	c.769C>G (hom)	p.(Leu257Val)	F	Yes	Lebanese	Yes	12 m		+		−	+		+	Visual loss; died following respiratory infection age 25 m	P1	[35]
44	*SDHD*	c.205G>A	p.(Glu69Lys)	F	No	Swiss	No	3 m	+	+		+	+	+		Seizures; secondary microcephaly; nystagmus	P1	[38]
		c.479G>T	p.(*160Leuext*3)†															
45	*SDHD*	c.275A>G (hom)	p.(Asp92Gly)	M	No	Irish	No	Prenatal			+						P1	[39]
46	*SDHAF1*	c.164G>C (hom)	p.(Arg55Pro)	F	Yes	Turkish		10 m	+	+		+				Leukoencephalopathy (no basal ganglia changes); spastic quadriplegia; growth delay; tetraparesis; cognitive impairment	P1	[40]
47	*SDHAF1*	c.164G>C (hom)	p.(Arg55Pro)	F	Yes	Turkish		9 m	+	+						Spasticity; growth delay; tetraparesis	P2	[40]
48	*SDHAF1*	c.164G>C (hom)	p.(Arg55Pro)	F	Yes	Turkish		10 m	+							Spasticity; growth delay; tetraparesis; irritability	P3	[40]
49	*SDHAF1*	c.164G>C (hom)	p.(Arg55Pro)	F	Yes	Turkish		10 m	+							Spasticity; growth delay; tetraparesis; irritability	P4	[40]
50	*SDHAF1*	c.164G>C (hom)	p.(Arg55Pro)		Yes			12 m				−		−			LD755 /P3	[41], [26]
51	*SDHAF1*	c.168G>C (hom)	p.(Gly57Arg)		Yes			18 m		+		−		+		Spasticity; tetraparesis; wheelchair dependent	P6	[26]
52	*SDHAF1*	c.168G>C (hom)	p.(Gly57Arg)		Yes			10 m		+		−		+		Spasticity; tetraparesis	P9	[26]
53	*SDHAF1*	c.169G>C (hom)	p.(Gly57Arg)	M	Yes	Italian	No	6 m	+			+				Spasticity; tetraparesis; deafness; growth delay	P5	[40]
54	*SDHAF1*	c.169G>C (hom)	p.(Gly57Arg)	F	Yes	Italian	Yes	10 m	+							Spasticity; growth delay; tetraparesis	P6	[40]
55	*SDHAF1*	c.169G>C (hom)	p.(Gly57Arg)	M		Italian		11 m	+							Spasticity; tetraparesis; irritability	P7	[40]
56	*SDHAF1*	c.170G>A (hom)	p.(Gly57Glu)	F	Yes	Palestinian		14 m	+							Spasticity	P4	[42]
57	*SDHAF1*	c.170G>A (hom)	p.(Gly57Glu)	F	Yes	Palestinian		4 m								Spasticity	P5	[42]
58	*SDHAF1*	c.3G>A (hom)	Start loss		Yes			12 m		+		−		+		Dysarthria; dysphonia; dysphagia; wheelchair dependent	P1	[26]
59	*SDHAF1*	c.3G>A (hom)	Start loss		Yes			11 m		+		−		+		Seizures; dysarthria; wheelchair dependent	P2	[26]
60	*SDHAF1*	c.22C>T (hom)	p.(Gln8*)	M	No	Norwegian		20 m								Leukoencephalopathy; spasticity; clumsiness	P3	[42]
61	*SDHAF1*	c.156C>A (hom)	p.(Tyr52*)		Yes			16 m		+		+		+		Spasticity; tetraparesis	P18	[26]

Abbreviations: dev - developmental; het - heterozygous variant; hom - homozygous variant; OA - optic atrophy; m - months; y - years; − symptom absent; + symptom present; empty cell signifies no data available. † reported as p.*164Leuext*3

Patients typically present with an onset of symptoms during childhood, consequent to bi-allelic pathogenic variants in one of the *SDHx* genes, consistent with an autosomal recessive inheritance pattern. The vast majority of clinically-affected individuals reported in the literature harbour genetic variants within the *SDHA* gene and present with a Leigh syndrome phenotype, clinically defined as a subacute necrotising encephalopathy [16]. Although less common, pathogenic variants involving the *SDHB*, *SDHD* and *SDHAF1* genes have also been reported (Table 1). Interestingly, there are yet to be reports to establish a link between primary mitochondrial dysfunction and genetic defects involving the structural subunit *SDHC* or the assembly factors *SDHAF2, SDHAF3* or *SDHAF4*. The results of the literature search into the genetic basis of mitochondrial complex II deficiency and the associated clinical phenotypes is presented below, stratified by causative *SDHx* gene.

#### 3.2.1 Pathogenic variants in the *SDHA* gene

Defects involving the flavoprotein-encoding *SDHA* gene are the most commonly reported cause of isolated complex II deficiency. A total of 17 unique pathogenic *SDHA* variants have been reported in the literature, in 32 clinically-affected subjects. *SDHA* is the only *SDHx* gene reported to date in which dominant pathogenic variants have been identified although most affected individuals harbour either homozygous or compound heterozygous pathogenic variants, consistent with autosomal recessive inheritance. Of historical note is the fact that pathogenic variants in *SDHA* represent the very first cause of Mendelian mitochondrial disease [21].

#### 3.2.2 Dominantly-inherited pathogenic *SDHA* variants

Only one variant - c.1351C>T p.(Arg451Cys) - has been reported to follow an autosomal dominant inheritance pattern; interestingly, it has been reported in the literature in five cases from two unrelated families. In the first family, two clinically-affected sisters (*Cases 1 and 2,*Table 1) presented similarly during the fourth decade of their lives with optic atrophy, ataxia and myopathy. Respiratory chain analyses of muscle biopsy revealed a 50% decrease in complex II enzyme activity [22]. The second family comprised three clinically-affected individuals - a father, son and daughter (*Cases 3, 4 and 5*, Table 1) [23]. All three individuals had symptoms presenting in childhood, though there was marked intra-familial variability in disease manifestation. The father presented with clumsiness and nystagmus subsequently developing ocular paresis, optic atrophy and ataxia, with cardiomyopathy diagnosed at the age of 15 years. Both children were clinically affected, his daughter succumbing to a severe cardiomyopathy at age 7 months, while his son was 30 years-old at last follow up and followed a similar disease course to that of his father, with early onset cardiomyopathy and subsequent bilateral optic atrophy. Respiratory chain analyses of patient skin fibroblasts revealed residual complex II enzyme activities of 51% (father), 42% (son) and 33% (daughter). Whilst a recessive, loss of function pathomechanism often underlies the clinical presentation, this is clearly not the case here and it is purported that an alternative mechanism must be necessary to explain the pathogenicity of the single heterozygous c.1351C>T p.(Arg451Cys) *SDHA* variant [22,23].

Western blot analysis of myoblasts from Family 1 (*Cases 1 and 2,*Table 1) and fibroblasts from Family 2 (*Cases 3–5,*Table 1) demonstrated no reduction in the steady state levels of the SDHA protein, suggesting that there was no effect on protein stability and implying a qualitative defect [22,23]. Complex II functions, ultimately, to generate electrons from the oxidation of succinate to fumarate, and transferring these electrons to ubiquinone. The crystal structure of the succinate dehydrogenase enzyme has been elucidated (PDB: 1ZOY) and studies have established that the specific residues critical for succinate binding are Thr308, Glu309 and Arg451, the latter being the residue implicated in these patients' dominant complex II deficiency [13,22]. The c.1351C>T p.(Arg451Cys) *SDHA* variant affects an evolutionarily conserved, critical residue within the succinate binding pocket (Fig. 1), it is consistent with pathogenicity but does not sufficiently explain the dominant mechanism; two working models have been proposed. First, Birch-Machin and colleagues studied the *Escherichia coli* homologue and found that the p.(Arg451Cys) substitution prevents the FAD cofactor forming a covalent bond with the critical histidine residue of SDHA, thus rendering the enzyme inactive [22].

A second hypothesis involves the succinate:ubiquinone oxidoreductase activity of complex II. A human model of p.(Arg451Cys) predicts that succinate is still able to bind to the remaining residues but a greater concentration of substrate is required due to its decreased affinity. Moreover, ubiquinone remains able to bind to complex II in spite of the p.(Arg451Cys) SDHA substitution but cannot be reduced, this would be consistent with a dominant pathomechanism [22,23].

#### 3.2.3 Recessively inherited pathogenic *SDHA* variants

With the exception of the dominantly acting c.1351C>T p.(Arg451Cys) *SDHA* variant, all other reported pathogenic *SDHA* variants are associated with autosomal recessive inheritance. Many variants are nonsense, frameshift or splicing variants that result in a null allele, but missense variants affecting key functional residues within the SDHA protein have been reported.

#### 3.2.4 Null *SDHA* variants

To date, eleven different null *SDHA* alleles have been reported in primary mitochondrial disease presentations. Only one, c.1A>C, involves the loss of the initiation methionine residue. The c.1A>C *SDHA* variant was identified in *trans* with a c.1571C>T p.(Ala524Val) *SDHA* missense variant (discussed in missense *SDHA* variant section) in a clinically-affected child who presented with Leigh syndrome (*Case 6,*Table 1); only marginal amounts of the c.1A>C-associated transcript were detectable, presumably utilising the in-frame methionine occurring at Met114, but would nevertheless be non-functional due to the absence of the N terminal residues of the protein which serve to target the apoprotein to the mitochondria [20].

Two nonsense variants have been reported, each in the heterozygous state and in *trans* with a second pathogenic *SDHA* variant. The first variant, c.356G>A, is predicted to cause a premature stop codon at p.(Trp119*). The c.356G>A p.(Trp119*) variant has been reported in just one case who presented at 5 months of age with developmental regression, nystagmus, seizures and hypsarrhythmia on electroencephalogram [EEG] (*Case 7,*Table 1); the variant was detected *in trans* with a pathogenic c.248C>T p.(Ala83Val)/p.(Ala83_Gln104del) variant [17,24]. Functional studies support nonsense-mediated decay of the associated *SDHA* mRNA transcripts which supports loss of function as the pathomechanism.

The second reported nonsense variant is a c.91C>T p.(Arg31*) *SDHA* substitution, identified in one patient who presented with Leigh syndrome and who died at 8 months of age (*Case 8,*Table 1); the variant was identified *in trans* with a c.565T>G p.(Cys189Gly) *SDHA* variant [17]. Functional studies confirmed the c.91C>T p.(Arg31*)*-*associated *SDHA* mRNA transcripts are subject to nonsense mediated decay, consistent with a loss of function pathomechanism. Interestingly, this variant was the first *SDHx* variant to be reported in association with both a primary mitochondrial disease presentation and tumour formation [17].

Four pathogenic frameshift *SDHA* variants have been reported in two compound heterozygous individuals. The first affected child presented at 4 years of age with encephalopathy and developmental regression following viral illnesses; MRI changes supported a clinical diagnosis of Leigh syndrome and bi-allelic c.117del p.(Asn40Thrfs*18) and c.221dup p.(Leu74Phefs*9) *SDHA* variants were identified (*Case 9,*Table 1) [25]. Unlike many other patients with complex II deficiency, this child was reported to be relatively well (physically and performing well at school) at the time of publication and had been prescribed various mitochondrial supplements including carnitine and ubiquinone (CoQ_10_).

The second patient (*Case 10,*Table 1) presented at 4 months of age with Leigh syndrome, and died from respiratory failure at 3 years of age. Genetic investigations identified compound heterozygous c.771_772del p.(Gly258Leufs*62) and c.1065_1260del p.(Gly356Serfs*2) *SDHA* variants [26]. The c.771_772del variant is situated immediately adjacent to the conserved dinucleotide splicing acceptor [26]. Multiple *in silico* prediction tools support abnormal splicing due to the c.771_772del *SDHA* variant through use of an alternative splicing acceptor, predicting a p.(Gly258Leufs*62) frameshift. The c.1065_1260del *SDHA* variant precisely removes the entire of exon 9, predicting a p.(Gly356Serfs*2) frameshift variant. The mRNA transcripts associated with the c.771_772del p.(Gly258Leufs*62) and c.1065_1260del p.(Gly356Serfs*2) *SDHA* variants are predicted to be degraded due to nonsense mediated decay on account of premature truncation.

Three intronic splice variants have been reported in *SDHA,* both occurring in the homozygous state. The first affected individual presented with seizures and leukodystrophy on MRI (*Case 11,*Table 1) [17]. Molecular genetic investigations confirmed a homozygous c.1065-3C>A *SDHA* variant in intron 8 that resulted in skipping of exon 9 (r.1065_1260del). Although this mRNA transcript was shown to be unstable and degraded, some wildtype *SDHA* mRNA transcript was present, consistent with the residual SDHA protein detectable in patient cell lysates following SDS-PAGE analysis and the complex II activity in patient fibroblasts, which was assayed at 24% of control levels [17].

The second *SDHA* splicing variant was identified in a child who presented at 16 months of age with ataxia and myopathy and whose cranial MRI displayed changes consistent with Leigh syndrome (*Case 12,*Table 1) [17]. Skeletal muscle biopsy demonstrated an isolated complex II deficiency, subsequently attributed to a homozygous c.64-2A>G *SDHA* variant affecting the invariant splicing acceptor dinucleotide preceding exon 2. Interestingly, this child was age 10 years at the time of reporting, despite the homozygous splicing variant.

Splicing investigations established that the c.64-2A>G *SDHA* variant also produced two different splicing products, but neither of which were wildtype [17]. The minor transcript, c.64_73del, utilises an alternative donor site within exon 2 (c.72_73) that predicts a p.(Trp22Cysfs*33) frame shift leading to a premature stop codon and mRNA transcripts that would be subsequently degraded [17]. The more abundant transcript resulted from the activation of a cryptic donor site within intron one, and the exonisation of c.64-54_64–1. This 54 bp insertion occurs in frame, and subsequently is anticipated to produce a stable protein [17]. It is possible that this SDHA protein may have some residual function given that the enzyme activity was greater in this patient, and that, at the time of the study, this patient remained alive at 10 years of age whereas the patient harbouring a homozygous c.1065-3C>A variant died before the age of 3 years [17].

The final pathogenic *SDHA* splicing variant, c.248C>T, was identified in a child who presented at 5 months of age with Leigh syndrome and seizures and was found to have hypsarrhythmia on EEG (*Case 7,*Table 1). Their other allele harboured a pathogenic c.356G>A p.(Trp119*) *SDHA* variant, discussed previously. The c.248C>T variant within exon 3 was originally reported as a p.(Ala83Val) missense variant, it was subsequently demonstrated to activate a cryptic donor site at c.247_248, resulting in skipping of the remaining residues of exon 3 before utilising the natural acceptor at exon 4, causing an in-frame p.(Ala83_Gln104) deletion [17,24]. The mis-spliced transcript is unstable and subject to nonsense mediated decay, but some residual full-length protein containing the p.(Ala83Val) variant was also detectable [17]. This is consistent with many other cases where one seemingly null allele is identified in *trans* with either a missense variant or a ‘leaky’ splicing variant where some residual wild-type transcript exists. There are two exceptions reported thus far: cases 9 [25] and 10 [26] were both found to harbour bi-allelic truncating variants although no functional data was presented to confirm the anticipated null mechanism. Particularly for Case 9, who certainly presents with a milder phenotype than would be expected with a bi-allelic null genotype, the possibility therefore remains that an unexpected mechanistic rescue may have occurred, for example in-frame exon skipping through activation of cryptic splicing enhancers or silencers.

#### 3.2.5 Missense variants in the *SDHA* gene

To date, only six missense variants in *SDHA* have been reported as a cause of mitochondrial disease, most of which are private mutations that have been reported in single families and occurring as compound heterozygous variants, perhaps indicating, that one variant in each pair must confer some residual function in order to be compatible with life.

A c.565T>G p.(Cys189Gly) *SDHA* variant was identified by Renkema and colleagues in *trans* with the c.91C>T p.(Arg31*) *SDHA* nonsense variant discussed previously (*Case 8,*Table 1) [17]. To briefly recap, the patient presented with Leigh syndrome and died at 8 months of age; assessment of respiratory chain activities confirmed an isolated complex II deficiency. SDS-PAGE and Western blotting of patient whole cell lysates suggested a moderate reduction in SDHA subunit expression and the highest levels of SDHA protein expression of all four cases presented. Complex II activity in fibroblasts was just 13% of control levels and was the most severe defect in the patients reported [17]. Together, these data support a qualitative impairment of complex II due to the c.565T>G p.(Cys189Gly) *SDHA* variant. Although it does not directly affect FAD binding, the replacement of a highly conserved cysteine residue with a glycine removes the cysteine side chain (and potential disulphide bond) and is likely to interfere with the configuration of the surrounding amino acids that interact with the FAD cofactor [17].

A heterozygous c.1571C>T p.(Ala524Val) *SDHA* variant was identified in *trans* with a c.1A>C variant (see *null SDHA variants*) in a previously discussed child who presented with Leigh syndrome (*Case 6,*Table 1) [20]. The p.(Ala524Val) substitution is predicted to contribute a methyl side chain to the SDHA protein, resulting in steric hindrance which is anticipated to affect the tertiary structure and consequently reduce complex II activity [20].

A further 19 patients have been reported who are homozygous for pathogenic variants involving one of two adjacent SDHA residues: c.1664G>A p.(Gly555Glu) [27] and c.1660C>T p.(Arg554Trp) [21]. Seventeen cases harbour a homozygous c.1664G>A p.(Gly555Glu) *SDHA* substitution, of which 15 are from two extended consanguineous Bedouin families. Each individual presented with dilated cardiomyopathy (DCM) within the first 8 months of life (*Cases 13–27,*Table 1) [27]; there was no clinical history of regression or MRI changes consistent with Leigh syndrome noted for any individual. Although there is little phenotypic diversity across the Bedouin individuals, two additional case reports serve to extend the phenotypic spectrum associated with the c.1664G>A p.(Gly555Glu) *SDHA* variant. The first case was a young boy who presented with developmental regression and MRI changes consistent with Leigh syndrome (*Case 28,*Table 1) [28] whilst the other was a young girl who presented aged 5 months in metabolic crisis following viral infection which resulted in rapid deterioration and demise from multi-organ failure (*Case 29,*Table 1) [29]. It is possible that Case 29 may have been too young for symptoms of Leigh syndrome (or indeed cardiomyopathy) to manifest. The presentation of fragile mitochondrial disease patients in metabolic decompensation following viral insult is not uncommon; the fever and catabolism associated with systemic infection increases demand and substrate availability for OXPHOS, but this is thwarted by the inefficiency of the process in these individuals ultimately creating an energy shortfall with consequences for cellular and organ function.

A pathogenic variant that affects the adjacent residue in SDHA has also been reported to cause complex II deficiency. The c.1660C>T p.(Arg554Trp) *SDHA* substitution was described in two sisters born to consanguineous Algerian parents who both presented at 10 months of age with developmental regression and whose MRI imaging were consistent with a clinical diagnosis of Leigh syndrome (*Cases 30 and 31,*Table 1) [21,30]; this case report represents the inaugural genetic diagnosis of Mendelian mitochondrial respiratory chain disease in man.

**Fig. 2:**
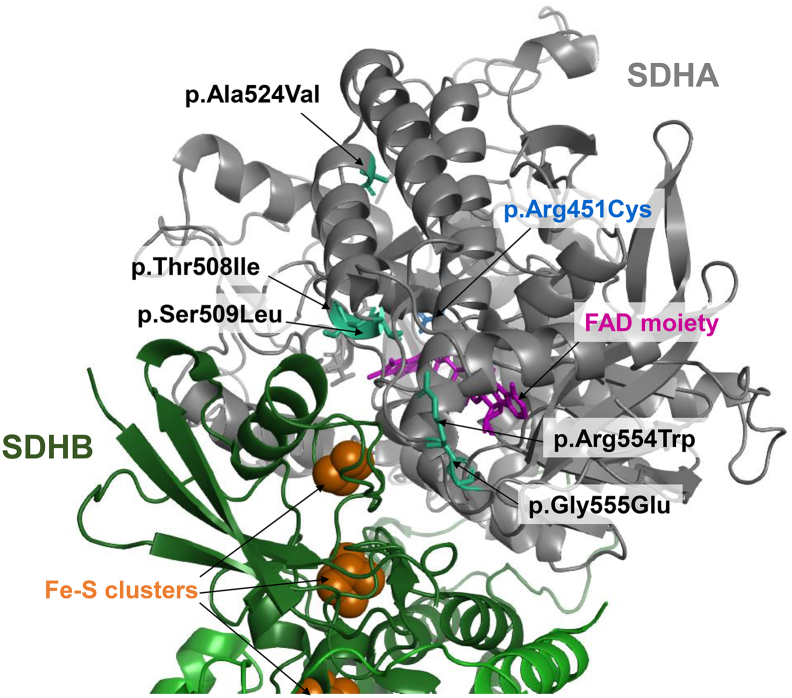
Structural modelling of reported *SDHA* missense variants onto the reported mammalian crystal structure of complex II (PDB: 1ZOY). The pathogenic missense variants, p.(Thr508Ile), p.(Ser509Leu), p.(Arg554Trp) and p.(Gly555Glu) variants are situated at the interface between the SDHA and SDHB subunits. Recessive *SDHA* variants are shows in black text, whilst the dominant p.(Arg451Cys) *SDHA* variant is shown in blue. The SDHA protein is shown in grey, whilst the SDHB protein is shown in green; the three iron‑sulphur clusters within SDHB are shown as orange clusters. (For interpretation of the references to colour in this figure legend, the reader is referred to the web version of this article.)

The adjacent Gly555 and Arg554 residues are situated within the N terminal domain of the flavoprotein [29], with structural modelling demonstrating their location at the SDHA-SDHB interface; the c.1660C>T p.(Arg554Trp) and c.1664G>A p.(Gly555Glu) variants are therefore likely to affect heterodimerisation (Fig. 2). Furthermore, Gly555 is shown to be of particular importance in an *Escherichia coli* homologue whereby a side chain of the corresponding residue, Ser495, is shown to form a hydrogen bond with an arginine residue on the SDHB subunit. Moreover, the p.(Arg554Trp) and p.(Gly555Glu) substitutions result in a more negative charge in the external loop of the flavoprotein, suggesting a shared pathomechanism, where modifications to either residue are capable of jeopardising the SDHA-SDHB interaction to the detriment of mitochondrial complex II assembly [29]. Defective complex II assembly was in fact confirmed in Cases 28 and 29, who presented with either Leigh syndrome or in acute metabolic crisis, respectively [28,29], where immunoblotting against the SDHA and SDHB subunits following Blue-Native PAGE revealed a marked decrease in the amount of fully-assembled complex II [28,29].

There is an enigmatic element relating to the pathogenicity of the c.1664G>A p.(Gly555Glu) *SDHA* variant in that a homozygous c.1664G>A p.(Gly555Glu) *SDHA* variant has also been detected in a healthy adult within the extended consanguineous Bedouin pedigree [27]. Structural modelling predicts the Gly555 to reside at the SDHA-SDHB interface and, it was hypothesized that he may harbour an amino acid substitution affecting the corresponding region of SDHB to circumvent a complex II assembly defect. Surprisingly, no candidate variants could be identified following sequencing of *SDHB*, *SDHD* or *SDHAF1* thus his normal phenotype cannot be explained.

To date, only one individual has been reported in the literature who harboured compound heterozygous *SDHA* missense variants involving adjacent amino acid residues: c.1523C>T p.(Thr508Ile) and c.1526C>T p.(Ser509Leu) [31]. The affected individual was a 3 month old boy who presented with dyspnoea and feeding difficulties (*Case 32,*Table 1). BN-PAGE analysis of enriched mitochondria revealed significantly decreased levels of fully assembled complex II whilst Western blotting demonstrated a near complete absence of SDHA expression in patient whole cell lysates [31]. Similar to the Arg554-Gly555 SDHA residues discussed previously, the Thr508 and Ser509 SDHA residues are also situated at the SDHA-SDHB interface and are therefore purported to affect heterodimer formation (Fig. 2). Although functional evidence supports defective SDHA as the cause of pathology, the c.1523C>T p.(Thr508Ile) *SDHA* variant has a minor allele frequency of 0.01% with one homozygous individual reported on gnomAD (https://gnomad.broadinstitute.org/); it is possible that the c.1523C>T p.(Thr508Ile) *SDHA* variant may behave like the Bedouin c.1664G>A p.(Gly555Glu) *SDHA* variant where there may be an additional factor contributing to penetrance. Alternatively, there may be a different aetiology - possibly the presence of an intronic variant *in cis* with the c.1523C>T p.(Thr508Ile) *SDHA* variant. This situation has been reported previously involving the *NUBPL* gene that encodes a complex I assembly factor, where an intronic variant was subsequently identified that co-segregated with the reported missense variant [32,33]. This report, together with the c.248C>T p.(Ala83Val) “missense” *SDHA* variant that in fact causes incomplete mRNA mis-splicing demonstrates the importance of cDNA analysis to confirm the pathomechanism of novel sequence variants.

### 3.3 Pathogenic variants in the *SDHB* gene

In contrast to *SDHA*, where there are numerous reports of a wide spectrum of pathogenic variants in clinically-affected patients, to date, only 6 manuscripts have been published that describe bi-allelic pathogenic *SDHB* variants. A total of 11 patients have been reported (*Cases 33–43*,Table 1), involving only five pathogenic *SDHB* variants, all of which are missense substitutions and inherited in an autosomal recessive manner. Each patient presented in infancy (age of onset ranging from birth to 19 months), with clinical presentations including leukoencephalopathy, cardiomyopathy and psychomotor regression (Table 1).

The most frequently reported variant is a c.143A>T point mutation predicting a p.(Asp48Val) missense substitution in *SDHB*. Of the nine patients that harbour this variant, seven are homozygous (*Cases 33–39,*Table 1) and two patients harbour an alternative pathogenic variant on the other *SDHB* allele (*Cases 40 and 41,*Table 1). There is phenotypic heterogeneity associated with the c.143A>T p.(Asp48Val) *SDHB* variant, with reported presentations including leukodystrophy, psychomotor regression and cardiomyopathy. Onset of symptoms occurs between birth and 18 months of age in all cases, although one report describes an asymptomatic sibling who was found during cascade testing to harbour a homozygous c.143A>T p.(Asp48Val) *SDHB* variant. There are no homozygous cases and only 11 carriers of the c.143A>T p.(Asp48Val) *SDHB* variant on gnomAD, which is consistent with pathogenicity; whether an additional insult is required for symptoms to manifest is uncertain – some children presented acutely following viral illness (e.g. cases 34 and 36) but this does not sufficiently explain the discrepancy [26,34].

Functional studies were undertaken to confirm the pathogenicity of the c.143A>T p.(Asp48Val) *SDHB* variant - modelling of the equivalent variant in yeast (p.Asn42Val) demonstrated a decrease of almost 50% in complex II activity compared to the wild type [31]. Moreover, a humanised yeast strain was generated (whereby Asn42 was substituted with Asp) which demonstrated no distinguishable difference in SDH activity compared to the wild type yeast, thereby confirming that the observed decrease in SDH activity was not due to the lack of homology at the locus [31]. Functional studies using patient muscle, fibroblasts and lymphoblasts revealed that the c.143A>T p.(Asp48Val) *SDHB* variant is associated with a considerable decrease in SDHB subunit expression, consistent with protein instability [31,35]. Patients also have a small reduction in SDHA expression compared to controls, suggesting that complex II assembly is also likely to be impaired [31,34]. Additionally, structural analysis predicts that the Asp48 residue is located at the 2Fe-2S binding domain and substitution of a negatively charged aspartate with an uncharged valine is likely to have a significant effect on the capacity for electron transfer within the catalytic iron-sulphur subunit of succinate dehydrogenase [31].

Although the pathogenic c.143A>T p.(Asp48Val) *SDHB* variant has been reported in many instances as a homozygous variant, it has also been reported in compound heterozygous cases. One patient (*Case 40,*Table 1) who presented with loss of motor skills secondary to leukoencephalopathy was found to harbour a c.143A>T p.(Asp48Val) *SDHB* variant in *trans* with a c.689G>A p.(Arg230His) *SDHB* variant [35]. His clinical course improved following riboflavin and CoQ_10_ therapy, until rapid deterioration lead to multiorgan failure and death at 1 year of age. The c.689G>A p.(Arg230His) *SDHB* variant has previously been identified as a driver of familial paraganglioma and pheochromocytoma development, although there is no evidence of increased susceptibility to tumorigenesis in the patient's family history. The c.689G>A p.(Arg230His) *SDHB* variant is only the second variant to be associated with both mitochondrial complex II deficiency and susceptibility to tumour manifestation [36].

The other reported case involving a heterozygous c.143A>T p.(Asp48Val) *SDHB* variant presented with febrile seizures and motor regression (*Case 41,*Table 1); they were found to harbour a c.308T>C p.(Met103Thr) *SDHB* variant on their other allele*.* The molecular genetic diagnosis was sought following identification of a characteristic MRI/MRS profile with elevated succinate levels identified in SDH-deficient patients [26]. The p.(Met103Thr) substitution is predicted to be located proximal to key catalytic residues within the 2Fe-2S iron‑sulphur cluster binding domain of SDHB [26].

Interestingly, a pathogenic variant involving the adjacent amino acid is also reported in a case of mitochondrial disease (*Case 42,*Table 1) [37]. The patient was an 18-month child who presented with developmental delay, hypotonia and ataxia, who was subsequently found to harbour a homozygous c.304G>A p.(Ala102Thr) *SDHB* variant [37]. The Ala102 residue is highly conserved and protein modelling predicts that it interacts with Gly99 within SDHB via a single polar contact [37]. The substitution of the alanine residue with threonine results in the formation of another polar bond with Cys189, altering the tertiary structure of the SDHB protein [37].

The final pathogenic *SDHB* variant reported in the mitochondrial disease literature was identified in a 12 month old child who presented with developmental delay and truncal hypotonia (*Case 43,* Table 1). The c.769C>G p.(Leu257Val) *SDHB* variant was identified in the homozygous state and involves a functionally important residue that is conserved from humans to yeast [35]. Western blot analysis using patient fibroblasts also showed similar results to those of the c.143A>T p.(Asp48Val) *SDHB* variant, whereby there is a clear reduction in SDHB expression along with a slight decrease in SDHA expression [35].

Unlike *SDHA*, where numerous null mutations have been reported to cause mitochondrial complex II deficiency, no null *SDHB* alleles have been reported to date. An alternative aetiology (bi-allelic *DARS2* variants) has been established for the clinically-affected individual in whom compound heterozygous c.541-2A>G and c.423+20T>A *SDHB* splicing variants were initially suspected; so, although the pathogenicity of the c.541-2A>G variant that affects the conserved donor sequence is not in question, it has yet to be implicated in mitochondrial disease [26].

### 3.4 Pathogenic variants in the *SDHD* gene

Just two patients have been reported in the literature to date with recessive pathogenic variants affecting the hydrophilic binding domains of complex II, both involving the SDHD subunit. The first patient presented at 3 months of age with developmental regression and suffered continuous neurological deterioration until death at the age of 10 years (*Case 44*,Table 1) [38]. She was found to harbour compound heterozygous *SDHD* variants, a c.205G>A p.(Glu69Lys) missense substitution and a c.479G>T p.(*160Leuext*3) variant (originally reported as p.*164Leuext*3) that affects the natural truncation codon and extends the SDHD protein by three residues [38]. The second case presented *in utero* with cardiac abnormalities identified on antenatal ultrasound (C*ase 45*, Table 1). He was found to harbour a homozygous c.275A>G p.(Asp92Gly) *SDHD* variant [39].

The c.205G>A variant predicts a p.(Glu69Lys) substitution at the strictly conserved Glu69 residue (conserved to *Escherichia coli*) within the first alpha-helical domain of SDHD. The c.479G>T changes the stop codon to a leucine residue (TAG>TAT), subsequently inducing the addition of two further amino acids (proline and phenylalanine) before the next in-frame stop codon halts translation thereby extending the predicted peptide by three amino acids in the fourth alpha-helical domain [38].

SDHD is an integral membrane protein with four alpha-helical domains localised within the inner mitochondrial membrane thus, these substitutions are likely to affect the ability of SDHD to integrate into the membrane correctly. This is supported by 10.13039/501100005016BN-PAGE analyses of both patients, whereby immunoblotting against the SDHA subunit was able to detect only very low amounts of the complex II holoenzyme in mitochondria enriched from the patient's muscle biopsy [38,39]. SDS-PAGE of the same mitochondrial preparation from Case 45 demonstrated a marked reduction in SDHD protein levels, and a moderate reduction in the levels of SDHA protein expression [39]. Pathogenicity of the variants was firmly established through a complementation experiment using patient fibroblasts; lentiviral transduction with the *SDHD* cDNA containing either the c.205G>A p.(Glu69Lys) or the c.479G>T p.(*160Leuext*3) *SDHD* variants was unable to rescue the phenotype, whereas transduction of the patient fibroblasts with the wild type *SDHD* cDNA transcript restored SDH activity [38].

The c.275A>G p.(Asp92Gly) variant affects a highly conserved Asp92 residue of SDHD and was also shown to cause a pronounced reduction in fully assembled complex II following BN-PAGE analysis of the patient's cardiac mitochondrial preparations [39]. Moreover, an SDHD yeast orthologue, SDH4, was used to further investigate the effect of the p.(Asp92Gly) substitution; the variant was modelled in yeast (the corresponding variant is p.Asp98Gly), and a severe growth defect was observed, similar to that of the SDH4 null mutant, corroborating its pathogenicity [39]. The Asp92 residue is predicted to be located within the N terminus of the second transmembrane helical domain of SDHD, a region likely to interact with heme *b*, therefore a mutation impacting this residue may disrupt heme *b* binding, or affect its stability.

### 3.5 Pathogenic variants in the *SDHAF1* gene

Of the four known SDH assembly factors, pathogenic variants resulting in mitochondrial complex II deficiency have only been reported in *SDHAF1*. The absence of pathogenic variants in *SDHAF2-4* might suggest that there are other proteins capable of compensating for defective SDHAF2, SDHAF3 or SDHAF4 but the task of incorporating Fe-S clusters into SDHB lies solely with SDHAF1 and this unique function makes it indispensable. Alternatively, the absence of pathogenic variants in these genes may be because they are crucial and defects are not compatible with life. To date, 16 patients have been documented in the literature with *SDHAF1-*related mitochondrial disease; a total of seven recessive, homozygous pathogenic variants were identified as causal across this cohort.

The most commonly reported variant is a c.164G>C transversion, predicting a p.(Arg55Pro) missense substitution; this variant has been reported in five clinically-affected individuals, four of whom belong to the same Turkish family; (*Cases 46–50*,Table 1) [40,41]. The age of presentation was 8–18 months, and the presenting clinical feature was acute psychomotor regression – this was precipitated by febrile illness for 3/4 cases [40]. Poor growth, severe spastic quadriplegia and cognitive impairment were noted as prominent features for each case in the family. The clinical features were very similar between the two families with common symptoms including psychomotor regression and spasticity [40].

A further seven patients have been reported to harbour one of three different homozygous pathogenic variants that involve the Gly57 SDHAF1 residue, either c.168G>C or c.169G>C which both predict a p.(Gly57Arg) substitution or a c.170G>A transition which predicts a p.(Gly57Glu) substitution [[26], [40], [42]].

Two unrelated individuals were reported to harbour a c.168G>C p.(Gly57Arg) *SDHAF1* variant (*Cases 51 and 52*,Table 1) [26] whilst three individuals (*Cases 53–55*,Table 1) have been reported to harbour a homozygous pathogenic c.169G>C p.(Gly57Arg) *SDHAF1* variant that results in the same amino acid substitution as that of cases 51 and 52. The c.169G>C p.(Gly57Arg) *SDHAF1* variant was presumed to be identical by descent; cases 53 and 54 were second cousins, and case 55 was presumed to be related on account of originating from the same rural region of Italy. Inspection of the DNA sequence reveals that c.168 is not a guanine nucleotide, but a cytosine, and does not form part of the Gly57 codon but represents the third nucleotide of Arg56; together, this suggests that cases 51 and 52 may in fact harbour a c.169G>C p.(Gly57Arg) *SDHAF1* variant, as identified in cases 53-55). All five children presented in a similar manner, aged between 6 and 11 months, with leukoencephalopathy, psychomotor regression and severe spastic quadriplegia which showed some response to riboflavin therapy [40]. The pathogenicity of the p.(Gly57Arg) missense variant was confirmed through modelling in yeast which demonstrated impaired complex II activity that was restored upon transformation with the wild-type *SDHAF1* cDNA [40]. Moreover, rescue of the biochemical defect was obtained following expression of wild-type *SDHAF1* cDNA in the p.(Gly57Arg) patient fibroblast cell line [40].

Another variant involving the Gly57 residue has been identified in two sisters (*Cases 56 and 57*, Table 1); both presented with motor regression, spasticity and developmental delay however, the eldest sister did not show symptoms until 14 months of age, in comparison to the younger sibling who presented at just 4 months of age [42]. They were found to have a complex II deficiency and a homozygous c.170G>A p.(Gly57Glu) *SDHAF1* variant was subsequently identified [42].

SDHAF1 is crucial for SDH assembly and has been shown to mediate the recruitment of Fe-S clusters to SDHB by interacting with the HSC20-HSPA9-ISCU complex via its two tripeptide Leu-Tyr-Arg (LYR) motifs - the highly conserved proximal LYR motif (Leu14-Tyr15-Arg16) interacts with HSC20, a component of the Fe-S complex whilst the distal LYR motif (Leu53-Tyr54-Arg55) interacts with SDHB [43]. The p.(Arg55Pro), p.(Gly57Arg) and p.(Gly57Glu) substitutions reported in cases 46–57 involve either the distal LYR motif directly (in the case of p.(Arg55Pro)) or the nearby Gly57 residue; it is therefore likely that the pathomechanism involves disruption of the interaction between SDHAF1 and the SDHB subunit.

A further four patients have been identified who harbour homozygous null *SDHAF1* alleles that either abolish the initiation methionine or introduce a premature stop codon. Firstly, two siblings presented at 11 and 12 months of age with dysarthria, dysphonia and dysphagia precipitated by injury or fever; they were subsequently found to each harbour a homozygous c.3G>A *SDHAF1* variant which is predicted to abolish the initiation methionine residue and therefore the translation of SDHAF1 (*Cases 58 and 59*, Table 1) [26]. The remaining pathogenic *SDHAF1* variants are homozygous truncating *SDHAF1* variants, reported in two patients. The first case presented at 20 months of age with increasing clumsiness and spasticity; he was found to harbour a homozygous c.22C>T p.(Gln8*) nonsense variant (*Case 60*,Table 1) [42]. This variant is predicted to truncate the SDHAF1 protein within the mitochondrial targeting sequence (MTS), obliterating both LYR motifs [42]. Interestingly, the entire coding sequence of *SDHAF1* is encoded by a single exon, meaning that it is likely to evade nonsense mediated decay. Without the MTS, it is difficult to reconcile how this variant is compatible with life. This patient, despite having a more severe SDH enzyme deficiency, presented with milder symptoms - clumsiness and spasticity - when compared with other patients harbouring missense *SDHAF1* variants [42]. Compensatory pathways to ameliorate the clinical presentation are possible, but the precise mechanisms remain unknown.

The second case was a 16-month-old child who presented with spastic tetraparesis and intellectual impairment, precipitated by fever (*Case 61*,Table 1) [26]. A homozygous c.156C>A p.(Tyr52*) nonsense variant in *SDHAF1* was identified. Again, the transcripts are likely to evade nonsense mediated decay, but are anticipated to truncate the SDHAF1 protein within the second LYR motif [26]. It would be interesting to determine whether there is any correlation between the length of the *SDHAF1* transcript and the clinical presentation, but at this stage there are too few patients in the literature to draw meaningful correlations.

## 4 Overlap between mitochondrial complex II deficiency and tumour susceptibility

There are just two reported pathogenic *SDHx* variants that are common to both mitochondrial complex II deficiency and tumour susceptibility, c.91C>T p.(Arg31*) in *SDHA* [17,44] and c.689G>A p.(Arg230His) in *SDHB* [35,45]. Despite their clear ability to cause either manifestation, there is no family history of the alternative clinical presentation in the families reported. This is not entirely unexpected given that a single germline pathogenic *SDHx* variant still requires a second hit to cause tumorigenesis, and conversely an individual with a single germline *SDHx* variant would have a 25% chance of having a child with bi-allelic *SDHx* variants only in the rare occurrence that their partner also harboured a pathogenic variant in the same gene. As with all rare diseases, the risk of this situation is very small but significant, having occurred for each affected individual not withstanding a *de novo* mutational event or mechanism such as uniparental isodisomy.

## 5 Treatment of mitochondrial complex II deficiency

There is no cure for complex II deficiency, though some cases collated here reported clinical improvement following riboflavin therapy [26]. Riboflavin (vitamin B2) is a component of flavin adenine dinucleotide (FAD) and flavin mononucleotide (FMN), cofactors of complexes I and II which are critical for electron shuttling [46]. The report of clinical improvement following riboflavin therapy is not unique to patients with an isolated complex II deficiency; a similar situation has been reported in other presentations including *ACAD9*-related mitochondrial disease [47]. Riboflavin is not the only therapeutic intervention that is available to patients with an isolated complex II deficiency and various other compounds have shown promise in the treatment of symptoms, including l-carnitine and ubiquinone. l-carnitine supplementation acts to restore levels of free carnitine which chaperone long chain fatty acids into the mitochondrion. These acylcarnitine esters are then oxidised to acetyl CoA which then enters the tricarboxylic acid (TCA) cycle and contributes downstream to OXPHOS. Increasing the contribution of the fatty acid oxidation pathway to the TCA cycle somewhat dampens the cells' dependence on the defective carbohydrate metabolism pathway for ATP production. Ubiquinone (CoQ_10_) is a ubiquitous and naturally-occurring antioxidant that is most abundant in the inner mitochondrial membrane where it transfers electrons between complexes I/III and II/III; increased bioavailability has been shown to reduce reactive oxygen species caused by stalled electron transfer [48]; it is commonly administered to mitochondrial disease patients and has shown some positive effect in the treatment of isolated complex II deficiency [25,35].

## 6 Concluding comments

Clinical diagnosis of mitochondrial complex II deficiency is determined by functional and/or genetic analysis. Historically, a “biopsy first” approach was routinely employed, where biochemical assessment of respiratory chain activities were undertaken using patient muscle biopsies. In the genomic era of mitochondrial diagnostics, it is more common to employ high throughput sequencing using DNA from a non-invasive EDTA blood sample to establish a genetic diagnosis. When ACMG classification [49] is consistent with a pathogenic variant, diagnosis is straightforward, but in cases where the pathogenicity is less certain then biopsy material (muscle and/or fibroblasts) are crucial to establishing the genetic diagnosis. In the absence of a cure, a genetic diagnosis facilitates genetic counselling and the provision of reproductive options for families.

Here we present a compendium of pathogenic *SDHx* gene variants that have been reported in the literature in patients with a biochemically confirmed isolated mitochondrial complex II deficiency. To date, 61 patients are reported in the literature to harbour 32 pathogenic variants in complex II genes; three structural subunit genes (*SDHA, SDHB* and *SDHD*) and one assembly factor gene (*SDHAF1*) (Table 1). All reported patients harbour homozygous or compound heterozygous variants, consistent with autosomal recessive inheritance, with the exception of one *SDHA* variant that follows an autosomal dominant inheritance pattern. Pathogenic variants arise consequent to null mutations in the form of nonsense, frameshift or splicing defects, or due to missense variants that affect highly conserved residues in regions of the proteins that are critical for binding to other subunits or substrates.

As with other mitochondrial diseases, there are no definitive genotype-phenotype correlations; the initial clear association between complex II deficient Leigh syndrome and bi-allelic pathogenic *SDHA* variants has been blurred by reports of *SDHA*-deficient cases presenting with cardiomyopathy [27,31], and conversely with complex II deficient Leigh syndrome cases subsequently found to harbour defects in other *SDHx* genes – *SDHAF1* [40,42], *SDHB* [31] and SDHD [38]. Across the complex II deficient cohort collated here, there are several very different presentations including Leigh syndrome, epileptic encephalopathy and cardiomyopathy. Some cases present following viral illness; this feature is not specific to mitochondrial complex II deficiency and occurs in many other mitochondrial disease presentations. The underlying pathomechanism is the ATP-consuming immune response, often with associated fever, which can tip the delicate balance in an asymptomatic individual sensitive to perturbed ATP homeostasis [50]. There is a varied age of disease onset from the prenatal period to adult life, with clinical heterogeneity and/or variable penetrance associated with some variants (e.g. *SDHA* c.1664G>A p.(Gly555Glu) or *SDHB* c.143A>T p.(Asp48Val)) which further complicates diagnosis and counselling. When looking at the natural history of mitochondrial diseases, the number of cases is often insufficient to permit meaningful genotype-phenotype correlations being drawn. There is a bias towards the publication of novel disease genes to the detriment of the description of large patient cohorts that may not contain novel disease-causing variants, meaning that the true numbers cannot be appreciated; the study and publication of large mitochondrial disease cohorts is crucial to overcoming this hurdle.

## Declaration of competing interest

None to declare.
